# Endotoxemia and Gastrointestinal Cancers: Insight into the Mechanisms Underlying a Dangerous Relationship

**DOI:** 10.3390/microorganisms11020267

**Published:** 2023-01-19

**Authors:** Vittoria Manilla, Natalia Di Tommaso, Francesco Santopaolo, Antonio Gasbarrini, Francesca Romana Ponziani

**Affiliations:** 1 Internal Medicine and Gastroenterology-Hepatology Unit, Fondazione Policlinico Universitario Agostino Gemelli IRCCS, 00168 Rome, Italy; 2 Translational Medicine and Surgery Department, Catholic University of the Sacred Heart, 00168 Rome, Italy

**Keywords:** lipopolysaccharide (LPS), toll-like receptor 4 (TLR-4), endotoxemia, intestinal barrier dysfunction, cancer

## Abstract

Lipopolysaccharide (LPS), also known as endotoxin, is a component of the membrane of gram-negative bacteria and a well-recognized marker of sepsis. In case of disruption of the intestinal barrier, as occurs with unhealthy diets, alcohol consumption, or during chronic diseases, the microbiota residing in the gastrointestinal tract becomes a crucial factor in amplifying the systemic inflammatory response. Indeed, the translocation of LPS into the bloodstream and its interaction with toll-like receptors (TLRs) triggers molecular pathways involved in cytokine release and immune dysregulation. This is a critical step in the exacerbation of many diseases, including metabolic disorders and cancer. Indeed, the role of LPS in cancer development is widely recognized, and examples include gastric tumor related to *Helicobacter pylori* infection and hepatocellular carcinoma, both of which are preceded by a prolonged inflammatory injury; in addition, the risk of recurrence and development of metastasis appears to be associated with endotoxemia. Here, we review the mechanisms that link the promotion and progression of tumorigenesis with endotoxemia, and the possible therapeutic interventions that can be deployed to counteract these events.

## 1. Introduction

Lipopolysaccharide (LPS), or endotoxin, is a component of the cell wall of gram-negative bacteria [1,2]. It is made by an inner hydrophobic portion called lipid A, a central core region constituted by oligosaccharides, and an external layer, the o-antigen, formed by repeating oligosaccharides units [3,4].

LPS is a strong stimulator of the immune system and represents one of the pathogens-associated molecular patterns (PAMPs) recognized by pattern recognition receptors (PRRs) expressed on the surface of innate immune cells [4,5]. Among PRRs, toll-like receptor 4 (TLR-4), a transmembrane protein from the TLRs family, specifically recognizes LPS [6]. TLR-4 is mainly expressed on myeloid cells [7] and binds LPS, forming a heterodimer with the myeloid differentiation factor-2 (MD-2) [8,9,10]; this interaction is favored by the co-receptor CD14, which works as both soluble and membrane-anchored protein, and by the LPS binding protein (LBP) [11,12]. TLR-4 triggers two different pro-inflammatory signals: the MyD88, leading to the activation of the nuclear factor-kappa B (NF-kB) with the production of several cytokines, and the TIR-domain-containing adapter-inducing interferon-**β** (TRIF) pathway, which promotes the synthesis of type I interferons (IFNs) other than the NF-kB pathway [13,14,15,16,17]. Therefore, LPS recognition has a pivotal role in host defense against infections. However, humans are continuously exposed to LPS derived from endogenous sources, such as the gut microbiota [18]. Defense mechanisms regulate the effect of LPS exposure by preventing an excessive inflammatory response that would lead to organ dysfunction, as occurs during sepsis and septic shock [19]. In physiological conditions, a healthy intestinal barrier prevents bacterial translocation (BT) and the passage of microbial products from the intestinal lumen into the systemic circulation [20]. The intestinal barrier is made of a complex interconnection of structures and elements, including epithelial and endothelial cells, which form respectively the intestinal epithelial (IEB) and the gut vascular barrier (GVB), mucus, antimicrobial peptides, and Immunoglobulines A (IgAs) [21,22]. The mucus layer is rich with defensive proteins and keep bacteria distant from the mucosa; in physiologic conditions, intestinal bacteria promote mucus secretion by goblet cells and influence mucus stratification through the production of short-chain fatty acids (SCFAs) from dietary fibers [23].

Both endothelial and epithelial cells are tightly bound together by intercellular adhesion proteins, called tight junctions (TJs), which are key regulators of intestinal barrier permeability and integrity [24]. Various dietary or lifestyle-related agents, such as alcohol, fructose and fat intake, or chronic diseases modulate intestinal permeability; in addition to the direct effect on the epithelium, these factors modulate the gut microbiota, inducing dysbiosis. This is the driving force of increased intestinal permeability, with consequent systemic inflammation related to BT and LPS overload, called endotoxemia [25,26,27,28,29,30,31,32]. The detrimental effects of unhealthy diets on gut barrier and microbiota also include a shift towards mucus-degrading bacteria, the increase in intestinal permeability, and bacterial translocation [33]. All the above-mentioned mechanisms lead to an exacerbated activation of the LPS-TLR-4-pathway, resulting in an amplified inflammatory response involving the whole organism, with acute and chronic harmful consequences. However, there is also the possibility that constant endotoxemia leads to the development of low-grade inflammation.

## 2. Endotoxemia and Low-Grade Inflammation

Unhealthy diets and metabolic disorders, such as obesity and type 2 diabetes mellitus, are associated with intestinal barrier dysfunction and dysbiosis, resulting in increased circulating LPS, a condition called metabolic endotoxemia (ME) [34,35,36,37]. The low-grade inflammation caused by ME, in turn, establishes a vicious cycle associated with worsening glucose and lipid metabolism, development of insulin resistance, and non-alcoholic fatty liver disease (NAFLD) [34,38,39], and generates a favorable environment for cancer cell transformation [40,41] and tumor progression [42]. The combination of high fat diet (HFD)-induced dysbiosis and metabolic shifts in microbial metabolites and cell energy pathways promote a pro-inflammatory environment in intestinal cancer [43,44,45], whereas dietary interventions seem to counteract this event [46]. For instance, SCFAs derived from dietary fermentation of dietary fibers are recognized as protective agents against chronic intestinal inflammation, and experimental studies showed that fiber intake ameliorate intestinal microbiota composition, glucose and lipid metabolism and body weight, which are all recognized factors implicated in tumorigenesis [47,48,49]. Both hyperglycemia and intestinal barrier impairment have a recognized oncogenic potential in colorectal cancer (CRC) development [50,51]. ME also increases reactive oxygen species (ROS) formation and DNA damage, favoring tumorigenesis process [50,51]. These results confirm that intestinal-deriving inflammation is a common denominator in metabolic disorders and cancer [52,53].

**Figure 1:**
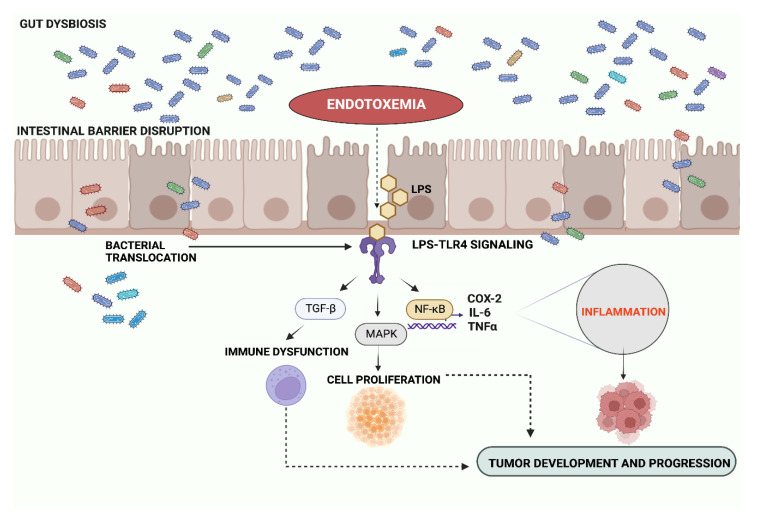
Pathways associated with endotoxemia and cancer risk. Gut dysbiosis and intestinal barrier impairment cause LPS translocation from gut lumen into the bloodstream, provoking endotoxemia and triggering systemic inflammation through LPS signaling in peripheral organs. During endotoxemia, LPS binding to TLR-4 activates several signaling pathways in targeted cells. The activation of the transcription factor NF-kB is associated with the production of pro-inflammatory cytokines that increase gut and systemic inflammation. Additionally LPS-TLR-4 signaling promotes the activation of cell cycle regulatory proteins and the secretion of cytokines that interfere with immune surveillance, sustaining a pro-oncogenic microenvironment in gastrointestinal cancers. Abbreviations: LPS: lipopolysaccharide; TGF-β: transforming growth factor beta; NF-kB: nuclear factor NF-kappa-B; MAPK: mitogen-activated protein kinase; TNF-α: tumor necrosis factor α; IL-6: interleukin 6; COX-2: cyclooxygenase-2. Created with BioRender.com.

Given the contribution of ME to increased oxidative stress [38], macrophage activation and tissue inflammation [54], it is tempting to speculate a direct connection between ME and carcinogenesis. Recently, an increased risk of cancer has been reported in patients with cardiovascular diseases and a persistent low-grade inflammation [55], but future studies are needed to clarify the exact role of ME in tumorigenesis in patients with metabolic disorders. The pathway that has been extensively studied and most closely connects tumor development and progression to endotoxemia is that of LPS-TLR-4-MD2 [56,57,58,59,60,61]. Figure 1 summarizes the main pathways associated with endotoxemia and cancer risk, which are also more extensively described in the following chapters.

## 3. Endotoxemia and Gastrointestinal Cancers

### 3.1. LPS and Esophageal Cancer

Esophageal cancer is often preceded by gastro-esophageal reflux disease (GERD) and Barrett’s esophagus (BE) precancerous lesion [62]. Compared to normal surrounding tissue, the microbiota associated with BE and esophageal adenocarcinoma (EAC) is mainly composed of gram-negative bacteria producing LPS, highlighting a strong inflammatory potential [63]. TLR-4 expression is significantly increased in biopsies from EAC, BE, duodenum, and reflux esophagitis compared to that from normal esophagus. Upon LPS stimulation, a TLR-4-dependent NF-kB activation is observed, followed by the production of IL-8 in all tissues but duodenum, as well as an increased expression of cyclooxygenase-2 (COX-2) in BE, suggesting a role in malignant transformation [64]. TLR-4 is also upregulated in esophageal squamous cell carcinoma (ESCC), and is correlated with poor differentiation and metastasis. LPS stimulation in this case decreases the expression of interleukin-10 (IL-10), while increasing the expression of tumor necrosis factor α (TNF- α) and transforming growth factor beta TGF-β, effects mediated by the ERK and p38 MAPK pathways [65]. Using an in vivo experimental model of liver metastasis secondary to esophageal human cancer, Rousseau et al. demonstrated that TLR-4 increases adhesive ability of esophageal cancer cells upon LPS stimulation. They also identified selectins and their ligands as key players in LPS-induced endothelial adhesion in vivo; in fact, after LPS stimulation, cells showed a two-fold increased adhesion to fibronectin and hepatic sinusoidal endothelium, with concurrent phosphorylation of p38. These effects were abolished by TLR-4 blockade or by p38 phosphorylation or selectin-selectin ligand-binding inhibition [66].

LPS can also induce lower esophageal sphincter dysfunction via the induction of nitric oxide synthase (iNOS), with the result of worsening gastroesophageal reflux disease (GERD) [67], and of delaying gastric emptying through the modulation of COX-2 transcription [68,69].

### 3.2. LPS and Gastric Cancer

*Helicobacter pylori* is universally recognized as a primary risk factor in the development of distal gastric carcinoma and gastric mucosal lymphoma [70]. *H. pylori* is a spiraliform gram-negative bacterium, able to produce various inflammatory particles, such as VacA, CagA and LPS [71]. However, LPS produced by *H. pylori* has always been considered less toxic than LPS produced by other bacteria, such as *Escherichia coli* [72] and enterohepatic *Helycobacter* subtypes [73]. Indeed, Hynes et al. tested the biological activity of the different types of LPS, finding that activity of *H. pylori* LPS, measured as endotoxin units, was about 100-fold lower than that of *E. coli* LPS [73]; this may explain why *H. pylori* establishes a chronic local infection, rather than a systemic inflammatory response [74]. Analyzing pathways activated by *H. pylori* LPS, Smith et al. demonstrated that *H. pylori* can induce responses in epithelial cells through Toll-like receptor 2 (TLR-2) and Toll-like receptor 5 (TLR-5), but not TLR-4, explaining the low pathogenicity of *H. pylori* [75]. Indeed, although TLR-4 and TLR-2 share a common MyD88-dependent signaling pathway, which results in the activation of nuclear transcription factors, such as NF-kB, TLR-4 can also induce the production of IFN-beta, leading to other effects not produced by TLR-2 agonists, such as the activation of iNOS [76]. On the contrary, some studies showed that TLR-4 mediates the interaction between *H. pylori* and host epithelial cells, initiating inflammation [77]. Yokota et al. recently further clarified the relationship between *H. pylori*, LPS and TLR-4 [74]. In a previous study [78], they showed that gastric cancer cell lines responded with a weak production of Interleukin-8 (IL-8) to *E. coli* LPS stimulation, due to a low cellular expression of TLR-4. Later, they demonstrated that pretreatment with *H. pylori* LPS substantially enhanced the reactivity of gastric cells to *E. coli* LPS in a dose-dependent manner. In particular, they distinguished three types of *H. pylori* LPS: a smooth LPS, with a strong antigenic epitope found in strains from gastroduodenal diseases (e.g., chronic gastritis and gastroduodenal ulcers); a smooth LPS with a weak antigenic epitope, isolated from gastric cancer patients; and a rough LPS. Rough LPS and smooth LPS, carrying the weakly antigenic epitope, showed a more potent *E. coli* LPS-enhancing activity when compared to smooth LPS, carrying the highly antigenic epitope. TLR-2 knockdown not only suppressed the production of IL-8, but also abolished the upregulation of TLR-4 by *H. pylori* LPS. They also demonstrated that TLR-4 upregulation by *H. pylori* LPS is mediated by the MEK1/2-ERK1/2 MAP kinase pathway, whose blockade not only reduces TLR-4 expression, but also gastric cell proliferation [74]. *H. pylori* also promotes TLR-4 cellular expression stimulating the transcriptional activation of myeloid differentiation factor 2 (MD-2), thus increasing the activation of NF-kB and IL-8 promoter [10]. The pathway LPS/TLR-4/MD-2 leads to CXC chemokine receptor 7 (CXCR7) expression in gastric mucosa, promoting cell proliferation and migration. LPS-mediated CXCR7 expression is blocked by TLR-4 or MD-2 knockdown, but also in MD-2 knockdown cells expressing only TLR-4. These findings suggest the important role of MD-2 in promoting cell proliferation and are consistent with the fact that the expression of MD-2 is significantly higher in gastric cancer tissue than in the surrounding tissue, and is correlated with lymphonodal metastases and TNM stage, predicting a worse prognosis and poorer survival [79]. Finally, *H. pylori* LPS, with its weak endotoxic activity, attenuates the cytotoxicity of mononuclear cells (MNCs) against gastric cancer cells, and downregulates perforin production in CD56+ natural killer (NK) cells. It also leads to the proliferation of regulatory NK cells producing IL-10, which diminishes the cytotoxic activity of the gastric epithelium in *H. pylori*-infected hosts. Thus, *H. pylori* LPS attenuates the cytotoxic activity of NK cells, which is the first line of anti-cancer immune defense [80].

### 3.3. LPS and Colorectal Cancer

Dysbiosis and gut barrier dysfunction are well-known factors implicated in the development of inflammation and colorectal cancer (CRC) [81]. Mice fed a high-fat diet (HFD) show an increased intestinal permeability and LPS serum levels, together with a greater tumor burden compared to their counterparts on conventional diet (CD). Fecal microbiota transplantation (FMT) from mice on HFD to mice on CD leads to an increase in tumor burden without significant body weight changes; on the contrary, antibiotic administration decreases tumor burden in HFD mice [43]. Wong et al. also demonstrated that FMT from patients with CRC can promote tumorigenesis in germ-free mice, increasing polyps number, cell proliferation, and the development of dysplasia and markers of inflammation [82]. LPS acts as a promoter of inflammation [83], which is a relevant risk factor for CRC occurrence; this has been strongly demonstrated in patients affected by ulcerative colitis, in whom the severity of inflammation is related to the risk of cancer development [84]. TLR-4 signaling, activated in response to LPS, induces, among others, COX-2 activation and the production of prostaglandin E2 (PGE2), which stimulates the expression on colonocytes and the release of amphiregulin, an epidermal growth factor receptor (EGFR) ligand involved in cell proliferation [85]. COX-2 expression in tumor-infiltrating macrophages is an early event in colon carcinogenesis, to the point that inhibition of COX-2 activity has been described as an effective chemopreventive strategy [86]. Not surprisingly, COX-2 is one of NF-κB target genes, and NF-κB expression is increased in stromal cells of human sporadic colorectal adenomas [87]. The existence of a strong relationship between gut microbiota, COX-2 and intestinal carcinogenesis is also proved by the synergic effect that aspirin and gut microbiota exert on CRC prevention. Aspirin modulates the gut microbiota by enrichment of probiotics, while gut microbiota depletion seems to boost the effect of aspirin on COX-2 reduction. In particular, aspirin administration reduces colorectal tumor number and load in APCmin/+ mice, which are predisposed to intestinal adenoma formation [88], and in mice given azoxymethane and dextran sulfate sodium treated with antibiotic, but not in mice with conventional microbiota. Plasma levels of aspirin are higher in mice receiving antibiotics than in controls; in particular, *Lysinibacillus sphaericus* is able to degrade aspirin, as also confirmed by the lower plasma levels of aspirin in germ-free mice fed *L. sphaericus* compared to germ-free controls [89]. In addition, the inverse correlation between aspirin dose and CRC development disappears proportionally with increasing *L. sphaericus* abundance. A possible explanation beyond the preventive effect of aspirin is the enrichment of the beneficial genera *Bifidobacterium* and *Lactobacillus* in the gut microbiota of treated mice, as well as the concomitant reduction in *Alistipes finegoldii* and *Bacteroides fragilis*, which are considered pathogens.

### 3.4. LPS and Hepatocellular Carcinoma

Approximately in 80% of cases, hepatocellular carcinoma (HCC) develops in conditions of advanced liver fibrosis or cirrhosis, which are characterized by prolonged inflammation and chronic liver injury [90]. BT is the hallmark of advanced chronic liver disease (ACLD), and is associated with the risk of complications [91]. Kupffer cells (KCs) are physiologically activated in response to LPS stimulation, producing cytokines in the attempt to clear a potentially harmful trigger; however, the consequent inflammatory response contributes to liver disease progression to the development of HCC [92]. As proof of concept, there is the fact that, despite a similar degree of intestinal barrier dysfunction, gut-derived inflammation appears to be greater in cirrhotic patients with HCC than in those without, as reported in patients with nonalcoholic fatty liver disease (NAFLD)-related cirrhosis [93]. Cytokines and chemokines significantly increased in NAFLD-related HCC are IL-8, IL-13, C-C motif chemokine ligand 3 (CCL3), CCL4 and CCL5, which are involved in fibrosis progression and hepatocarcinogenesis and, interestingly, are produced by an LPS-dependent mechanism [93,94,95,96]. In a multistage rat model of hepatocarcinogenesis obtained after chronical exposure to diethylnitrosamine (DEN), LPS plasma levels increase during tumor progression [97]. Different strains of gram-negative bacteria, such as *E. coli,* show an increased growth in stools and cecum at week 10 after DEN treatment, with a reduced abundance of benign bacteria (e.g., *Lactobacillus*, *Bifidobacterium* and *Enterococcus*); finally, subsequent probiotics administration is able to attenuate DEN-induced hepatocarcinogenesis [98]. Dapito et al. also highlighted that prolonged treatment with low-dose LPS significantly increased HCC development and showed that not only gut sterilization, but also genetic TLR-4 inactivation, decreases HCC development by 80%, playing a key role in the late stages of hepatocarcinogenesis [99]. In contrast to the above-mentioned studies, in a model of obesity-related HCC, Yoshimoto et al. reported a slight increase in HCC development in mice lacking the TLR-4 gene, also finding an increased percentage of gram-positive bacterial strains indigenous to humans and rodents after feeding a HFD. They confirmed their results, showing that treatment with vancomycin, which targets gram-positive bacteria, is able to prevent HCC development [100].

LPS is capable of activating KCs via the LBP/CD14/TLR-4 pathway and is responsible for the production of multiple pro-inflammatory cytokines, such as tumor necrosis factor (TNF)-alpha and IL-6 in the liver [92,101]. Binding of LPS to LBP enables translocation into the nucleus of NF-kB, which is usually retained in the cytoplasm by a family of inhibitory proteins called IκB kinases; this allows the transcription of multiple genes related to the inflammatory response, as well as the function of hepatocytes, KCs and hepatic stellate cells (HSCs) [102,103,104]. NF-kB can also inhibit apoptotic events through iNOS induction [105]. Therefore, the role of NF-kB in hepatocarcinogenesis seems to be ambiguous, showing different functions in different cellular compartments. On one hand, NF-kB protects hepatocytes from cell death when inflammatory and immune responses are initiated; on the other hand, it promotes HSCs and hepatic myofibroblasts (HMFs) survival during fibrogenic responses [104]. Watson et al. demonstrated the ability of NF-kB to inhibit HMFs apoptosis by blocking its pathway through a recombinant cell-permeable IKB alpha super repressor protein [106]. The IKK complex is activated by LPS and is capable of phosphorylating IkB protein, thus promoting NF-kB nuclear translocation [107]; its importance is widely recognized, to the point that it has been used as a therapeutic target to prevent liver fibrosis [108]. In fact, autocrine angiotensin II (AT-II) is capable of stimulating IKK-mediated phosphorylation of NF-κB subunit RelA at serine 536 (P-Ser536-RelA), promoting fibrosis, as happens in renal and cardiac fibrotic diseases [109]. Constitutive P-Ser536-RelA is a feature of HMFs found in livers exposed to chronic injury and is critical for nuclear transport and transcriptional activity of NF-kB [110]. Inhibition of angiotensin II, angiotensin II receptor type 1 (AT1), or IKK blocking P-Ser536-RelA results in the apoptosis of HMFs. Rodents with fibrotic liver treated with captopril, an angiotensin-converting enzyme (ACE) inhibitor, or sulphasalazine, an IKK inhibitor, leads to loss of P-Ser536-RelA-positive HMFs and fibrosis regression [109]. In human liver samples, an increased number of P-Ser536-RelA-positive cells is also associated with fibrosis development, which regresses after exposure to the AT-II receptor blocker (ARB) losartan [110]. The relationship between LPS and AT-II is also supported by the involvement of gut dysbiosis in arterial hypertension induced by angiotensin-II [111], with a proved reduction not only of blood pressure levels, but also of gut permeability after ACE inhibition; in particular, a complete reversal of fibrotic area and thickness of the muscularis layer, with a significant increase in the length of villi, was observed after treatment [112]. Based on these findings, different ongoing clinical trials are trying to investigate the role of ARB on fibrosis prevention [113,114,115].

### 3.5. LPS and Pancreatic Cancer

The exposure of pancreatic cells to LPS has been recently linked to the development of pancreatic ductal adenocarcinoma (PDAC). Indeed, expression of TLR-4 has been documented in PDAC cell lines; exposure to LPS resulted in the upregulation of 3083 genes in AsPC-1 and 2584 in PANC-1 cell lines, respectively, and the pathway mostly affected was I3K/Akt/mTOR [116]. Increased expression of TLR-4 in pancreatic cancer cells and tissues was also confirmed by Liu et al., showing also how pancreatic cancer cells migration is increased after treatment with LPS, and is accompanied by the inhibition of two tumor suppressor genes: the phosphatase and tensin homolog (PTEN) and MAP2K4. PTEN and MAP2K4 are targets of miR-181a, which decreases after LPS treatment [117]. In CRC, miR-181a directly targets PTEN and negatively regulates its expression; miR-181a gene transcription is dependent on the activation of NF-κB [118], another significant component of LPS/TLR-4 signaling pathway. Notably, NF-kB RelA transcription factor is constitutively activated in PDAC cells [119] and is responsible for the production of multiple chemokines, such as COX-2, IL-6, and IL-1 [120], whose role in PDAC development has been recognized in different experimental studies [121,122,123].

## 4. Endotoxin and its Role in Cancer Progression and Development of Metastasis

The role of endotoxin is not limited to the initial phases of carcinogenesis, but seems to be involved in cancer progression and development of metastases [124]. LPS enhances the adhesion of tumor cells to the endothelium through NF-kB activation [124], and increases the expression of laminin, an extracellular matrix (ECM) protein critical in the early stages of organogenesis and in the regulation of cell adhesion, migration, proliferation and differentiation, as well as in the development of new vessels [125]. As previously explained, TLR-4 is the main receptor involved in LPS inflammatory pathway [6]; in tissue samples from patients with HCC, TLR-4-positive HCC cells show stem-like properties with enhanced invasion and migration potential. Moreover, increased expression of TLR-4 in HCC tissue is strongly associated with early recurrence and poor survival. These results indicate that TLR-4 may act as a cancer stem cell marker, promoting mechanisms of tumor invasion and migration [90]. TLR-4 activation can also induce a dysfunction of the immune microenvironment, favoring cancer progression and being associated with worse patient survival in different solid tumors [126]. In fact, among cytokines released upon TLR-4 activation, TGFβ, VEGF, CCL-2 and IL-10 can induce CD4+CD25+Foxp3+ regulatory T cells (Tregs) [127], which exert an immunosuppressive effect in the tumor microenvironment [128]. TLR-4 also promotes the polarization of M2 macrophages (alternatively activated [129]), which is related to tumor relapse after chemotherapy [130], lymphatic metastasis and a poor prognosis in CRC [131,132]. Exploiting the TLR-4/MyD88 pathway, LPS enhances the invasive potential of PDAC in vitro [133]. Moreover, especially in the perioperative phase of CRC treatment, LPS increases urokinase plasminogen activator (u-PA) expression, which exerts a determinant role in regulating cancer stem cells-ECM interaction [134]. Interestingly, the infective risk in the perioperative phase following CRC surgical treatment is strictly associated with an increased risk of recurrence, both in local or at distant sites, and characterized by increased levels of endotoxemia [135,136,137]. One of the possible reasons behind infection-associated risk of disease progression is the formation of neutrophil extracellular traps (NETs) against bacteria [138]. NETs are made of loose DNA molecules interconnected in a web-like structure, histones, myeloperoxidase (MPO), neutrophil elastase, cathepsin G, and lactoferrin [139]. In murine models of infection, it has been demonstrated that NETs could capture circulating lung carcinoma cells, just like microbial cells, allowing them to reach distant organs, such as the liver, and form metastases [140]. Wang et al. documented an increased risk of relapse and metastatic spread in patients experiencing infective complications after CRC surgical treatment, finding an increased number of NETs in this group of patients. In experimental mouse models of CRC, intraperitoneal injection of LPS was associated with NETs formation; conversely NETs formation was absent in control mice, and their number was decreased after treatment with DNAse I. An increased number of molecules implicated in the activation of MAPK pathway (e.g., TLR-9, p-p38, p-Stat) was observed in cells cultured in media derived from LPS-stimulated neutrophils. The same molecules significantly decreased after treatment with DNase I and were reduced in TLR-9 deficient cells, with a concomitant decrease in tumor cells migration and invasion, suggesting TLR-9 as a possible therapeutic target in preventing recurrences [138].

## 5. Blocking LPS/TLR-4/M2 Cascade: Therapeutic Approaches

The alteration of the delicate gut microbiota homeostasis, primary endogenous source of LPS [18], and gut barrier dysfunction [27,33] are the driver of endotoxemia [141]. At the beginning of this cascade, there are well-known oncogenic risk factors, such as alcohol consumption, unhealthy diets, obesity, and other chronic inflammatory conditions [25,26,27,28,29,142].

**Figure 2:**
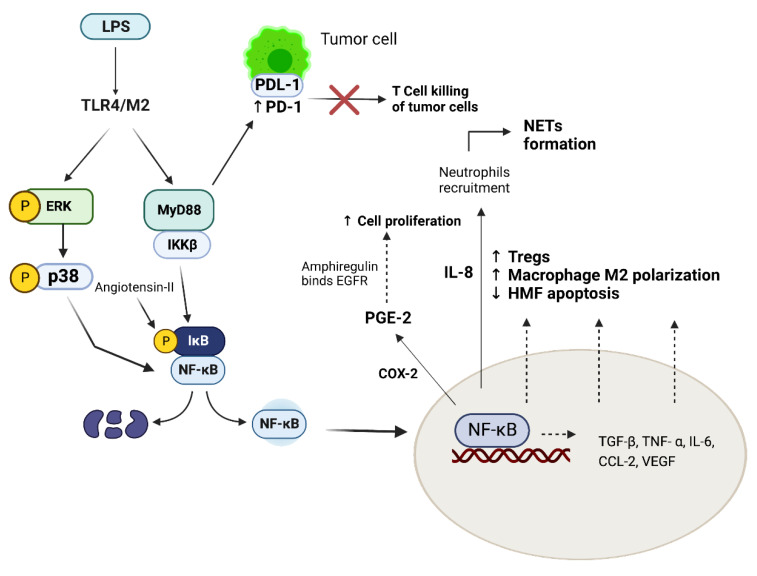
Molecular pathways involved in tumor development and progression. LPS, through TLR-4, activates MyD88 and MAPK/ERK/p38 pathways, both capable of promoting NF-kB nuclear translocation—in particular, through MyD88 pathway, IKKB phosphorylates IkB, which detaches from NF-KB, allowing nuclear translocation. This mechanism is favored by autocrine Angiotensin-II. Once in the nucleus, NF-kB activation leads to the transcription of multiple cytokines. COX-2 promotes PGE2 production, which stimulates the release of amphiregulin, an EGFR ligand involved in cell proliferation. IL-8 is involved in neutrophils recruitment, with subsequent NETs formation. Moreover, NF-kB activation leads to a reduced apoptosis of HMF and HSC, increases the number of Tregs and enhances M2 macrophages polarization, altering the immune tumor microenvironment, thereby promoting tumor cells immune escape. LPS/TLR-4/MD2 pathway also increases PD-1/PDL-1 bond, blocking T cell killing of tumor cells. Abbreviations: LPS: lipopolysaccharide; TLR-4: toll-like receptor 4; MAPK: mitogen-activated protein kinase; IKKB: inhibitor of nuclear factor-κB (IκB) kinase (IKK) complex; NF-kB: nuclear factor NF-kappa-B; COX-2: cyclooxygenase-2; PGE2: E2 prostaglandin; EGFR: Epidermal growth factor receptor; IL-8: Interleukin-8; NETs: neutrophil extracellular traps; HMF: hepatic myofibroblasts; HSC: hepatic stellate cells; TGF-β: tumor growth factor- β; TNF-α: tumor necrosis factor α; IL-6: interleukin 6; CCl-2: chemokine (C-C motif) ligand 2; VEGF: vascular endothelial growth factor; PD-1: programmed death-1; PDL-1: programmed death ligand-1; Tregs: regulatory T cells. Created with BioRender.com.

Once in the bloodstream, LPS interacts with toll-like receptors, in particular TLR-4, activating NF-kB and leading to an inflammatory and immune-modulating response, which is not only limited to the induction of the tumorigenesis, but is also involved in cancer progression and metastasis development [143]. Mechanisms and molecules involved in this process are described in Figure 2.

Several studies have investigated how to inhibit this multistep process at different points, starting from BT. Indeed, dietary interventions can reduce LPS translocation [144]: microbiota production of SCFAs from dietary fibers regulates intestinal inflammation [145], reinforces TJs integrity in case of intestinal dysbiosis [146] and inhibits NF-kB activity [147], while alcohol consumption and HFD are known to be associated with the development of serrated colorectal polyps, recognized as precancerous lesions [148]. Moreover, in the animal model of NASH, high-fructose feeding is associated with an increased risk of HCC development [149]. Probiotics help to modify the gut microbiota, promoting the growth of beneficial bacteria [150], and are an alternative source of SCFAs [151]. They seem to reduce LPS expression, intestinal inflammation and tumor size in CRC [152]. Among them, *Akkermansia muciniphila* has the ability to reduce LPS expression by reduction of LBP [153] and inhibition of NF-kB [154], improving metabolic endotoxemia [155]. *Parabacteroides diastasonis,* a gram-negative bacterium, is capable of reducing tumor incidence in murine models of HFD-induced CRC, and showed the ability to antagonize the TLR-4-MyD88 pathway [156]. Antibiotic treatment also decreases intestinal LPS overload [157]; in murine models of colitis-associated CRC, antibiotics and the TLR-4-blocking molecule Resatorvid [158] are able to inhibit inflammation, decreasing TLR-4 signaling and TLR-4+ tumor-infiltrating macrophages [159]. However, in a mouse model of methionine/choline-diet-induced NASH, antibiotics administration was reported to have negative effects [160], and a negative impact on HCC immunotherapy is described in different studies [161,162,163], so further evidence is needed to elucidate the role of antibiotics in gut microbiota modulation and tumor development. TLR-4 inhibition of host anti-tumor activity is also controversial, because, if it is true that tumor progression involves TLR-4-mediated production of pro-inflammatory and immunosuppressive cytokines, TLR-4 antagonists reduce pro-inflammatory response, but also compromise host immunity [164]. Instead, MD-2 inhibitors not only reduce tumor progression, but also show a potential action in decreasing CRC spread to the lung [60]. As previously discussed, targeting AT-II pathways or IKK to block P-Ser536-RelA is a promising tool to prevent liver fibrosis [109,110,113,114,115]. Among ARB, Losartan has also been studied in combination with Lenvatinib, sensitizing liver cancer cells to its cytostatic and angiostatic effects, thus allowing reduction of the Lenvatinib dose [165]. In NASH fibrosis, the combination of an ARB and rifaximin was studied, showing a stronger inhibitory effect when compared to that conferred by a single agent. This can be related to a complementary effect, as ARB inhibit HSCs, whereas rifaximin improves intestinal permeability through improving intestinal tight junction proteins (ZO-1) [166]. Another possible combined treatment involves the use of aspirin and probiotics in CRC, as already described [89]. Analyzing new therapeutic targets, IL-6 is certainly associated with liver tumorigenesis in early stages; recent studies investigated the synergistic effect of IL-6 inhibition in combination with inhibitors of PI3K/Akt/mTOR signaling cascade and immunotherapy in cancer treatment, finding stronger effects on HCC progression of the combination IL-6 antibody and NVP-BEZ235 treatment [167]. Il-6 blockade seems to improve the immunosuppressive environment when also combined with programmed death-1/programmed death ligand-1 (PD-1/PD-L1) inhibitors [168]. PD-L1 is an immunosuppressive molecule produced in response to TLR-4/MyD88 signaling cascade, which favors immune escape in tumor cells [169,170]; its blockade has a positive impact in decreasing PDAC growth when associated with LPS reduction [169]. Nowadays, there is also a growing interest in discovering the role of microRNA (miRNA) in normal cells, as well as in disease processes. miRNAs are a family of small non-coding RNAs which regulate the expression of various oncogenes or tumor suppressor genes [171]. miR-146a-5p reduces the production of IL-1β, IL-6 and TNF-α after HSC incubation with LPS [171] and can decrease the production of a-smooth muscle actin (ASMA) [172], which is synthesized by HSCs upon LPS induction [173] and is associated with an immunosuppressive microenvironment and poor HCC prognosis [174,175].

## 6. Conclusions

In recent years, many pieces of the intricate pathways linking BT, endotoxemia and gastrointestinal carcinogenesis have been unraveled, all having inflammation as a common thread. Whichever organ of the digestive tract is affected, the available data clearly indicate a role in tumor initiation, but also in tumor progression to metastasis. Several studies have revealed the controversial role of antibiotics and the valuable help provided by probiotics, additionally suggesting old and new molecules to be used as therapeutic targets to block cancer initiation and progression. All these findings reinforce the idea that a major effort is needed to accurately define all the pathways through which LPS is involved in carcinogenesis and to identify the true kay player in the never-ending battle against cancer development and progression.

## Data Availability

Not applicable.
